# Assessing the endocrine disrupting potentials and genotoxicity in environmental samples from Taiwanese rivers

**DOI:** 10.1186/s41021-019-0140-9

**Published:** 2019-12-30

**Authors:** Pei-Hsin Chou, Chien-Hsun Chen, Kuang-Yu Chen, Fung-Chi Ko, Tsung-Ya Tsai, Yi-Po Yeh

**Affiliations:** 1 Department of Environmental Engineering, National Cheng Kung University, 1, University Road, Tainan, 701 Taiwan; 2 Graduate Institute of Marine Biology, National Dong Hwa University, 2, Houwan Road, Pingtung, 944 Taiwan; 3 National Museum of Marine Biology and Aquarium, 2, Houwan Road, Pingtung, 944 Taiwan

**Keywords:** Endocrine disrupting potential, Genotoxicity, Yeast-based reporter gene assays, Rec-assay, Polycyclic aromatic hydrocarbons

## Abstract

**Background:**

Surface waters receive a variety of organic pollutants via wastewater discharge, and sediment represents a sink for hydrophobic contaminants. In this study, we used in vitro yeast-based reporter gene assays and a *Bacillus subtilis* Rec-assay to examine the occurrence of endocrine disrupting activities and genotoxic potentials in samples collected from three Taiwanese rivers. Levels of 51 polycyclic aromatic hydrocarbons (PAHs) in muscles of fish captured from same rivers were also analyzed to assess in vivo pollution of PAHs.

**Results:**

Antagonist activities for androgen receptor and retinoid X receptor (RXR) were detected in river water extracts at environmentally relevant concentrations., and sediment extracts exhibited RXR agonist, RXR antagonist, and genotoxic potentials concurrently. Σ16 PAHs in fish muscles ranged from 44.9–242.4 ng g^− 1^ dry weight, representing 38 to 59% of the total 51 PAHs concentrations, and methylated PAHs of low molecular weight PAHs were often detected as well.

**Conclusion:**

Taiwanese river sediment samples concomitantly exhibited RXR disrupting potentials and genotoxic activities, whereas RXR agonist and antagonist activities were simultaneously detected in several dry-season sediment extracts. PAH levels in fish muscles were categorized as minimally polluted by aromatic compounds, nonetheless, the presence of methylated PAHs in muscles samples may be of concern owing to the higher toxic potentials than their parent compounds.

## Background

Concerns over the presence of anthropogenic pollutants in the environment have been raised with the development of trace analytical techniques. Among numerous contaminants, conventional organic pollutants such as polycyclic aromatic hydrocarbons (PAHs) have drawn much attention owing to their environmental persistence and various adverse effects to organisms [1]. Along with the routine monitoring of target contaminants, it is also important to carry out mixture toxicity assessment since a diversity of pollutants are concomitantly present in the aquatic environment. Nowadays, the progress in establishing recombinant cell bioassays enables time- and cost-efficient toxicity evaluation of substances showing similar toxic responses in environmental matrices. In vitro assays such as Ames test, SOS/*umu* test, micronucleus test, or comet assay are well known as useful tools for mutagenicity and genotoxicity screening of environmental samples [2–9]. In vivo measurement of DNA adducts in fish has been used as biomarkers of genotoxicant exposure as well [10–13]. For example, fish collected from PAH-contaminated sites showed significantly higher mean levels of DNA adducts in hepatic tissues than those from a relatively non-polluted site in Québec, Canada [12]. A high amount of DNA adducts was also detected in fish caught from French Atlantic Coast two months after an oil spill [13].

In addition to persistent organic pollutants, emerging contaminants such as endocrine disrupting compounds (EDCs) are also of environmental concern. EDCs are chemicals that may act like (anti-)hormones or disrupt the synthesis and metabolism of hormones to alter the normal function of endocrine systems [14, 15]. Contaminants that interact with androgen receptor (AR) and estrogen receptor (ER) represent an important category of EDCs owing to their possible interference with reproductive function. Bioanalytical tools such as the yeast estrogen/androgen screen assays, the MCF-7 cell proliferation assay, and the chemically activated luciferase expression assays using rat/mouse cells transfected with AR/ER-regulated luciferase reporter genes have been broadly used to examine estrogenic/androgenic activities in surface water and wastewater effluents [16–19]. While sex hormone mimics in the environment have been comprehensively investigated, concerns are raised over new classes of EDCs. A battery of in vitro bioassays have thus been developed and used for detecting contaminants capable of binding to different receptors, such as thyroid hormone receptor (TR), glucocorticoid receptor (GR), mineralocorticoid receptor (MR), progesterone receptor (PR), retinoid X receptors (RXRs), etc. [20–24].

Over the past few decades, high levels of organic pollutants have been detected in Taiwan’s aqueous environment, suggesting a potential threat to aquatic biota [25–29]. Herein, mixture effects of a diversity of EDCs and genotoxicants present in Taiwanese river samples were investigated using yeast-based reporter gene assays and *Bacillus subtilis* Rec-assay, respectively. PAH levels in fish were also measured as a biological indicator for river pollution. Our objectives were to explore the occurrence of different toxic potentials in Taiwan’s aquatic environment and to assess the degree of PAH contamination in fish collected from downstream of Taiwanese rivers.

## Methods

### Reagents

Reagents used in this study were all of analytical grades. Dimethyl sulfoxide (DMSO), methanol, acetone, hexane, and anhydrous sodium sulfate were obtained from Merck (USA). Glucose, galactose, 17*β*-estradiol (17*β*-E2), 4-hydroxytamoxifen (OHT), dihydrotestosterone (DHT), flutamide (FLU), triiodothyronine (T3), 9-*cis* retinoic acid (9*c*RA), chlorophenol red-*β*-D-galactopyranoside (CPRG), *o*-nitrophenyl*-β*-D-galactopyranoside (ONPG), and 4-nitroquinoline-1-oxide (4-NQO) were purchased from Sigma (USA).

### Collection of river water, sediment, and fish samples

**Fig. 1:**
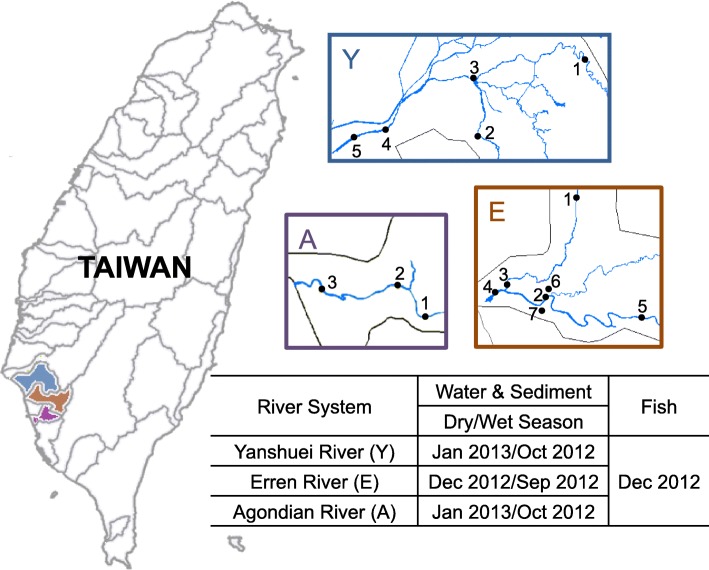
Sampling locations, site numbers, and schedule for water, sediment, and fish samples collected from Yanshuei River (Y), Erren River (E), and Agondian River (A) of Taiwan

Water, sediment, and fish samples were collected from three rivers that flow through densely populated areas in southern Taiwan, including Yanshuei River (5 sites, Y1–Y5, sediment of Y4 was not available), Erren River (7 sites, E1–E7), and Agondian River (3 sites, A1–A3) (Fig. 1 and Additional file 1: Table S1). Fish were captured downstream of each river (Y5, E4, A3) with the help of local fishermen, and were transported to laboratory on ice as soon as possible. Fish samples collected were mainly pollution-tolerant benthic species that could survive at low dissolved oxygen concentration (Additional file 1: Table S2).

### Pretreatment of river water, sediment, and fish samples

Each water sample (1 L) was filtered through 0.60 μm glass fiber filters (Advantec, Japan) and solid phase-extracted using two conditioned Sep-Pak® Plus Environmental C18 Cartridges (Waters, USA). Each cartridge was eluted with 3 mL of methanol and 1 mL of DMSO, and the eluates were concentrated by vacuum evaporation (CVE-3100, EYELA, Japan) and redissolved in DMSO.

Each sediment (Sed) sample was homogenized to pass through a 20 mesh sieve following freeze-drying in a freeze dryer (FDU-1200, EYELA, Japan) for at least 24 h. Then, 10 g of each pretreated sample was soxhlet-extracted with anhydrous sodium sulfate-added hexane:acetone (1:1, 200 mL) solution for 24 h. After extraction, the extract solution was added with 0.5 g anhydrous sodium sulfate and was evaporated to less than 3–5 mL by rotary evaporation (EYELA, Japan). Extractant was further purified by passing through an alumina oxide column and desulfurized by adding activated copper. The final extractant was concentrated using a purified nitrogen stream to 1 mL of DMSO (concentration: 10000 mg Sed-equivalent mL DMSO^− 1^) [26].

Fish dissection was carried out following species identification and length/weight measurement (Additional file 1: Table S2). Muscles of four fish from Y5 (Y5F1–Y5F4), 2 fish from E4 (E4F1, E4F2), and 4 fish from A3 (A3F1–A3F4) were collected and subjected to PAH analysis. Fish muscles were freeze-dried and extracted with dichloromethane in an accelerated solvent extractor (ASE-300, Dionex, USA). Lipid content was determined by gravimetric method and then was removed as stated in previous research [30].

### Endocrine disrupting activity evaluation

ER, AR, RXR, and TR disrupting activities of river samples were investigated using yeast-based reporter gene assays carried out as described in previous studies [22–24, 26–28]. In short, an overnight recombinant yeast culture was mixed with a sample, a negative control (DMSO), or a positive control (17*β*-E2, DHT, 9*c*RA, T3, OHT, FLU) in a 96-well microplate, and was incubated at 32/30 °C for 72/18 h. Each experiment was carried out in triplicate. The medium was pre-mixed with 0.3 nM 17*β*-E2 and CPRG solution for testing ER antagonist activity, and the medium was added with 25 nM DHT/9*c*RA/T3 for testing AR/RXR/TR antagonist activities. The concentrations of 17*β*-E2, DHT, 9*c*RA, and T3 used in the antagonist assays were approximately the half maximal effective concentrations (EC_50_) in the agonist assays (Additional file 1: Figure S1). The cell suspension was mixed with ONPG solution after sample exposure and was further incubated at 37 °C for 1 h for analyzing AR/RXR/TR disrupting activities. The absorbances at 620 nm (A_620_), 540 nm (A_540_), 595 nm (A_595_), and 405 nm (A_405_) were measured by a microplate absorbance spectrophotometer (xMark, Bio-Rad, USA) for calculating agonist/antagonist activity (ER agonist activity: fold induction of DMSO (FI_DMSO_) = [(A_540_)_SAMPLE_–[(A_620_)_SAMPLE_–(A_620_)_DMSO_)]]/(A_540_)_DMSO_, ER antagonist activity: FI_0.3 nM E2_ (%) = [(A_540_)_SAMPLE_–[(A_620_)_SAMPLE_–(A_620_)_0.3 nM E2_]]/(A_540_)_0.3 nM E2_ × 100%, AR/RXR/TR agonist activity: FI_DMSO_ = (A_405_/A_595_)_SAMPLE_/(A_405_/A_595_)_DMSO_, AR/RXR/TR antagonist activity: FI_25 nM DHT/9*c*RA/T3_ (%) = (A_405_/A_595_)_SAMPLE_/(A_405_/A_595_)_25 nM DHT/9*c*RA/T3_ × 100%). FLU, 17*β*-E2, and OHT equivalent concentrations (EQ) were calculated using the concentration-activity curves of corresponding standard compounds (Additional file 1: Figure S1).

### Genotoxicity testing

The *Bacillus subtilis* Rec-assay was applied to evaluate genotoxic potential in river sediment samples [31]. In brief, the survivals of a recombination proficient (Rec+) strain H17 (arg^−^, trp^−^, recE^+^) and a recombination deficient strain (Rec–) M45 (arg^−^, trp^−^, recE^−^) were compared to assess possible genotoxic effects of tested samples. 4-NQO and DMSO were used as positive and negative controls, respectively. Experiments were carried out as previously described [25], and genotoxicity was calculated as R_50_, which is the ratio of median inhibitory concentrations (IC_50_) of Rec+ and Rec– (R_50_ = IC_50, Rec+_/IC_50, Rec–_).

### Analysis of PAH levels in fish muscles

Concentrations of 51 non-substituted and methylated PAHs in fish muscle samples collected at Y5, E4, and A3 were determined as described [32] using a Varian 3800 GC/Saturn 4000 ion trap mass spectrometry (GC–MS) equipped with a 30 m Varian VF-5 ms capillary column (i.d.: 0.25 mm, film thickness: 0.25 μm) under the selected ion monitoring mode. Perdeuterated PAH surrogates (d_*8*_-napthalene, d_*10*_-fluorene, d_*10*_-fluoranthene, d_*12*_-perylene) were added to the procedural blanks, whereas d_*10*_-acenaphthene, d_*10*_-phenanthrene, d_*12*_-benz[*a*]anthracene, d_*12*_-benzo[*a*]pyrene, and d_*12*_-benzo[*g,h,i*]perylene were used as internal standards and added to each sample prior to analysis. The method detection limits (MDLs) and recoveries of each PAH are listed in Additional file 1: Table S3, whereas PAH levels were not corrected for surrogate recoveries.

## Results and discussion

### Endocrine disrupting potentials in river water and sediment extracts

**Fig. 2:**
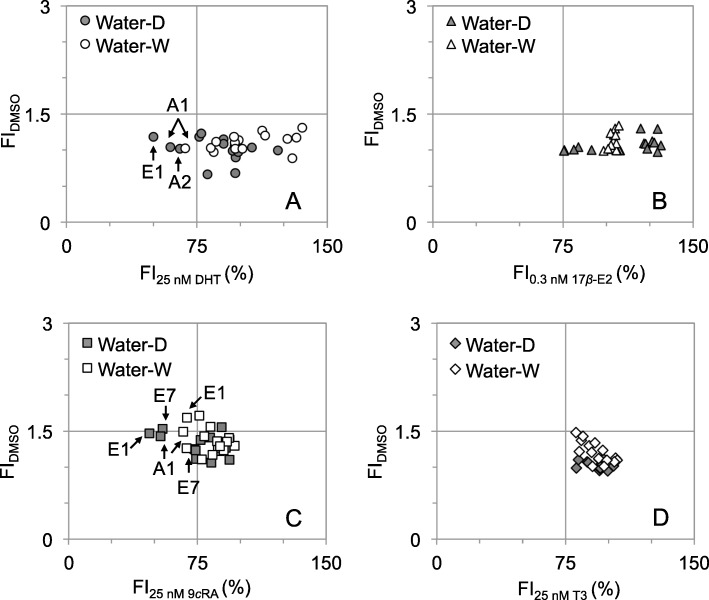
**a** AR **b** ER **c** TR **d** RXR agonist and antagonist activities elicited by dry-season river water extracts (Water-D) and wet-season river water extracts (Water-W) at environmentally relevant concentrations

Figure 2 shows the AR/ER/RXR/TR disrupting activities elicited by river water extracts at environmentally relevant concentrations. More than 13 and 30% of the river water extracts demonstrated significant antagonist activities for AR and RXR (Fig. 2a and c, lower-left region, FI_25 nM DHT/9*c*RA_ < 75%), respectively, whereas ER/TR disrupting activities were only detected in 10-fold and 5-fold concentrated water extracts (Additional file 1: Figure S2). The highest ER agonist, ER antagonist, and AR antagonist activities were found in the dry-season water extracts of E3, Y2 (17*β*-E2- EQ: 34.6 ng L^− 1^, OHT-EQ: 65.5 μg L^− 1^) and the wet-season water extract of A1 (FLU-EQ: 3377.5 μg L^− 1^), respectively. Although the values of 17*β*-E2-EQ, OHT-EQ, and FLU-EQ were lower than what have been detected in other sites of Taiwanese rivers [27, 28], our results suggested the pseudo-persistence of EDCs interfering with AR/ER signaling in river waters of Taiwan.

**Fig. 3:**
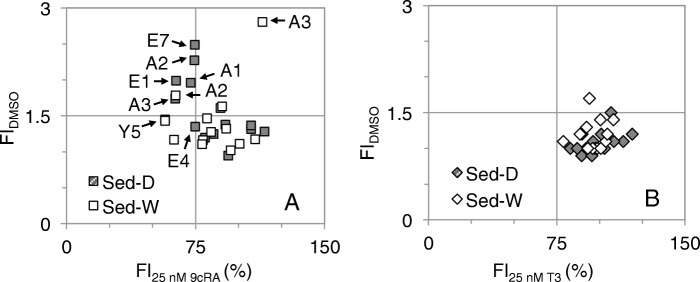
**a** RXR **b** TR agonist and antagonist activities elicited by dry-season river sediment extracts (Sed-D) and wet-season river sediment extracts (Sed-W) (concentration: 100 mg Sed-equivalent mL DMSO^− 1^ for agonist activity measurement and 50 mg Sed-equivalent mL DMSO^− 1^ for antagonist activity measurement)

RXR agonist and antagonist activities were found in dry- and wet-season sediment extracts with the detection frequencies of 32 and 36%, respectively (Fig. 3a), whereas TR disrupting activities were rarely detected in sediment extracts (Fig. 3b). In particular, all dry-season sediment extracts of Agondian river (A1–A3), E1, and E7 elicited RXR agonist and antagonist activities concomitantly (upper-left region, FI_DMSO_ > 1.5 and FI_25 nM 9*c*RA_ < 75%, Fig. 3a), while the co-existence of agonistic/antagonistic substances may lead to lower estimation of the disrupting potentials. Additionally, dry-season sediment extracts collected at Y5, E4, and A3 also exhibited significant RXR antagonist activities, suggesting that RXR antagonists may accumulate in downstream river sediments.

In the past decades, diverse anthropogenic substances have been identified as potential RXR agonists or antagonists. For example, organotins used as ingredients in antifouling paints have been well-known as potent ligands to activate human RXR [33], whereas tributyltin, tetrabutyltin, tripropyltin, tricyclohexyltin, and triphenyltin were shown to induce ligand-dependent transactivation of *Thais clavigera* RXR [34]. RXR agonist activities of various monohydroxylated polychlorinated biphenyls congeners (OH-PCBs), in particular tri- or tetra-chlorinated OH-PCBs were also identified recently [35]. By contrast, the plastic additive bisphenol A (BPA) and its chlorination by-products have been demonstrated to exhibit RXR antagonist activities [36]. Several statins (fluvastatin, pitavastatin) and nonsteroidal anti-inflammatory drugs (*R*-etodolac, sulindac) were recognized as RXR antagonists as well [37–39]. In the aquatic environment of Taiwan, the contaminants mentioned above or their parent compounds (i.e. PCBs) have been extensively detected [27–29, 40, 41]. For instance, butyltins and phenyltins have been found in Taiwan’s river sediment (ND–465 and ND–787 ng g^− 1^) and fish muscle samples (11–6860 and ND–1458 ng g^− 1^), where as phenyltins were dominant in freshwater environments possibly owing to illegal pesticidal use [40]. The RXR agonist/antagonist activities in Taiwanese river samples may be partially attributed to the occurrence of these recalcitrant contaminants.

### Genotoxicity in river sediment extracts

**Fig. 4:**
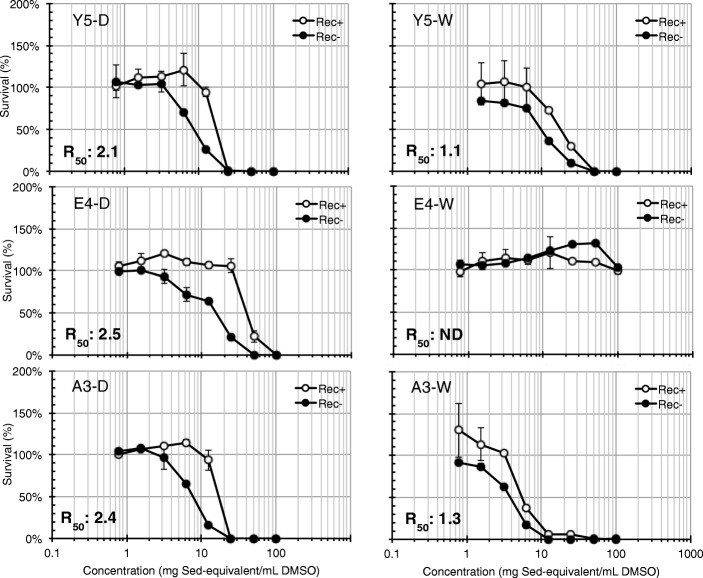
Concentration-survival rates of Rec+ and Rec– strains exposed to river sediment extracts of Y5, E4, and A3 (left: dry-season sediment extracts (D), right: wet-season sediment extracts (W)). R_50_ > 1.5 indicates significant genotoxic activity (ND: no detectable bacterial inhibition, IC_50,Rec+_ and IC_50,Rec–_ were greater than 100 mg Sed-equivalent mL DMSO^−1^)

Rec-assay analysis revealed that significant genotoxic activities (R_50_ > 1.5) were detected in dry-season sediment extracts of Y5, E1, E4, E7, A1, A3, and wet-season sediment extract of E7 (Fig. 4 and Additional file 1: Figure S3). Figure 4 shows the concentration-survival curves of Rec+ and Rec– strains exposed to different concentrations of river downstream sediment extracts (Y5, E4, A3), and the sediment extracts of A3 exhibited the lowest IC_50_ values for both strains. It is also noteworthy that the dry- and wet-season sediment extracts of A2 (Additional file 1: Figure S3) and wet-season sediment extracts of Y5 and A3 (Fig. 4) may be polluted cytotoxic substances owing to the detection of cytotoxicity instead of genotoxicity. Our previous work also reported that genotoxic activities were found in the sediment extracts of Y5, E1, E4, E7, and A1–A3 collected during different years [25], suggesting the constant input of genotoxicants into these sites.

Table 1 lists the RXR/TR disrupting potentials and genotoxic activities found in the sediment extracts analyzed in this study. It is interesting that the sediment extracts showing genotoxicity concurrently exhibited RXR disrupting activities. Several RXR agonists and antagonists, such as previously mentioned organotins or BPA and its analogs have been reported as environmental genotoxicants [42, 43]. BPA is a typical EDC widely known to possess estrogenic, anti-androgenic, and genotoxic potencies. Its concentrations in river waters and suspended solids of E1 have been reported to be as high as 725 and 12.3 μg L^− 1^, respectively [27]. Although BPA concentrations in sediment extracts are not analyzed in this study, its hydrophobic nature may lead to high accumulation in river sediments, which may contribute to the RXR disrupting activities and genotoxic potentials found in sediment extracts.

**Table 1:** RXR/TR agonist activity (FI_DMSO_), antagonist activity (FI_25 nM 9cRA/T3_ (%)), and genotoxicity (R_50_) of sediment extracts of Yanshuei River, Erren River, and Agodian River of Taiwan

Site	RXR Disrupting Activity				TR Disrupting Activity				Genotoxicity R_50_	
	FI_DMSO_^*1^		FI_25 nM 9*c*RA_ (%)^*2^		FI_DMSO_^*1^		FI_25 nM T3_ (%)^*2^			
	Dry^*3^	Wet^*4^	Dry	Wet	Dry	Wet	Dry	Wet	Dry	Wet
Y1	1.2	1.5	81%	82%	1.0	1.2	83%	89%	ND^*5^	ND
Y2	0.9	1.1	94%	79%	1.0	1.3	87%	92%	ND	ND
Y3	1.4	1.6^*7^	92%	91%	1.1	1.4	96%	101%	ND	ND
Y5	1.4	1.2	**57%**	**62%**	1.2	1.4	101%	108%	**2.1**	1.1
E1	**2.0**	**1.6**	**64%**	89%	1.0	1.0	92%	99%	**1.8**	> 1.25^*6^
E2	1.3	1.1	115%	101%	1.0	1.0	103%	98%	ND	ND
E3	1.2	1.2	85%	110%	1.1	1.1	114%	104%	ND	ND
E4	1.4	1.3	**75%**	84%	1.2	1.0	119%	99%	**2.5**	ND
E5	1.3	1.0	107%	95%	0.9	1.0	90%	96%	ND	ND
E6	1.4	1.2	107%	79%	1.0	1.0	96%	93%	ND	ND
E7	**2.5**	1.4	**75%**	**57%**	1.5	1.2	108%	105%	**1.8**	**2.9**
A1	**2.0**	1.3	**72%**	93%	1.1	1.0	108%	101%	**1.8**	ND
A2	**2.3**	**1.8**	**74%**	**63%**	0.9	1.1	95%	79%	1.3	1.2
A3	**1.7**	**2.8**	**63%**	114%	1.2	1.7	90%	94%	**2.4**	1.3

*1: sample concentration was 10 mg Sed-equivalent mL DMSO^−1^; *2: sample concentration was 5 mg Sed-equivalent mL DMSO^−1^; *3: dry season; *4: wet season; *5: no detectable bacterial inhibition, IC_50, Rec +_ and IC_50, Rec-_ were greater than the highest concentrations in the dilution series; *6: IC_50, Rec +_ was greater than the highest concentration in the dilution series; *7: Numbers in boldface are FI_DMSO_ > 1.5, F_I25 nM 9cRA/T3_ (%) < 75%, and R_50_ > 1.5

### PAHs in fish captured downstream of Taiwanese rivers

**Fig. 5:**
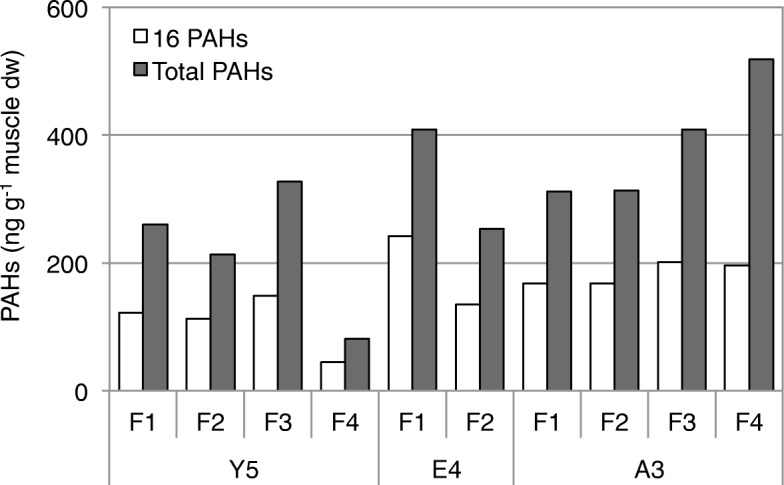
Summed concentrations of 16 USEPA priority PAHs (16 PAHs) and total summed concentrations of 51 PAHs (Total PAHs) in muscle samples of fish collected at Y5, E4, and A3

Levels of 51 PAHs (including 16 USEPA priority PAHs) in muscles of fish samples collected from Y5, E4, and A3 were investigated in this study. As shown in Fig. 5, concentrations of Σ16 PAHs in fish muscles varied from 44.9–242.4 ng g^− 1^ dry weight (dw) (11.0–52.4 ng g^− 1^ wet weight (ww)), constituting 38 to 59% of the total PAHs concentrations (81.0–518.5 ng g^− 1^ dw, 19.9–88.4 ng g^− 1^ ww). The National Oceanic and Atmospheric Administration has classified the concentrations of aromatic compounds in tissue samples into four categories, which are not contaminated (< 10 ng g^− 1^ ww), minimally contaminated (10–99 ng g^− 1^ ww), moderately contaminated (100–1000 ng g^− 1^ ww), and highly contaminated (> 1000 ng g^− 1^ ww) [44]. Total PAH levels in the muscle samples analyzed in this study could be categorized as minimally polluted by PAHs. Moreover, the average PAH concentration of four fish from A3 were significantly higher than that of fish from Y5 (*p* < 0.05, *t*-test), which corresponded to the higher genotoxicity/cytotoxicity detected in the sediment extracts of A3.

Levels of Σ16 PAHs were further compared to those detected in fish muscle samples from other countries. The concentrations of Σ16 PAHs in muscles of fish from Taiwanese rivers were comparable to those in fish tissues from Ghana (58–453 ng g^− 1^ dw) but higher than those in fish tissues from Italy (1.3–13.3 ng g^− 1^ dw), Spain (3–40 ng g^− 1^ dw), Nigeria (8.8–26.1 ng g^− 1^ dw), and Canada (11–116 ng g^− 1^ dw) [45–50]. Among individual PAHs, higher mean concentrations of low molecular weight PAHs (LMW-PAHs, 2- to 4-rings), such as naphthalene, phenanthrene, dibenzothiophene, and pyrene were detected at 75.0 ± 52.7, 38.2 ± 20.2, 19.9 ± 11.3 ng g^− 1^, and 17.7 ± 9.3 ng g^− 1^ dw, respectively. Our results were similar to other studies that LMW-PAHs originated from petrogenic sources were identified as the predominant compounds in a diversity of fish species collected from different countries [45–50].

Several methylated derivatives of legacy and heterocyclic PAHs were found at higher frequencies and levels as well, such as 2-methylnaphthalene (14.3 ± 7.8 ng g^− 1^ dw), 1,6-dimethylnaphthalene (9.4 ± 4.6 ng g^− 1^ dw), 2-methylphenanthrene (11.0 ± 6.3 ng g^− 1^ dw), 1-methylphenanthrene (9.1 ± 5.3 ng g^− 1^ dw), 1-methylanthracene (10.6 ± 5.6 ng g^− 1^ dw), and 4,6-dimethyldibenzothiophene (25.3 ± 14.6 ng g^− 1^ dw). Methylated PAHs have been shown to elicit potent disrupting activities for ER and aryl hydrocarbon receptor, and their hydroxymethyl derivatives have also been suggested to be potential carcinogens [51–53]. More research should be undertaken to assess the potential risk of methylated PAHs in edible fish species.

## Conclusion

Sediment samples collected from three polluted Taiwanese rivers concomitantly exhibited RXR disrupting potentials and genotoxic activities. By contrast, river water samples only showed AR/RXR antagonist activities at environmentally relevant concentrations. Noteworthily, RXR agonist and antagonist activities were simultaneously detected in several dry-season sediment extracts, suggesting higher disrupting activities were present in these samples. PAH levels in fish muscle samples fall into the category of minimally polluted by aromatic compounds, however, the detection of methylated PAHs may be of concern owing to the higher toxic potentials than their parent compounds.

## Supplementary information

**Additional file 1:** **Table S1.** Basic information of Yanshuei River, Erren River, and Agondian River^*1^. **Table S2.** Biological parameters of the fish captured at downstream of Yanshuei River, Erren River, and Agondian River. **Table S3.** Method detection limits (MDLs) and recoveries of target PAHs investigated in this study. **Figure S1.** Concentration-activity curves of agonist/antagonist compounds for AR (DHT/FLU), ER (17*β*-E2/OHT), RXR (9*c*RA), and TR (T3). **Figure S2.** (A) AR (B) ER (C) RXR (D) TR agonist/antagonist activities elicited by 10−/5-fold concentrated dry-season river water extracts (Water-D) and wet-season river water extracts (Water-W). **Figure S3.** Concentration-survival rates of Rec+ and Rec– strains exposed to genotoxic standard 4-NQO and dry−/wet-season (D/W) sediment extracts of E1, E7, A1, and A2.

## Data Availability

Data sharing is not applicable to this article as no datasets were created or analyzed during this study.

## Abbreviations

AR: Androgen receptor
EDCs: Endocrine disrupting compounds
ER: Estrogen receptor
GC–MS: Gas chromatography–mass spectrometry
PAHs: polycyclic aromatic hydrocarbons
RXR: Retinoid X receptor
TR: Thyroid hormone receptor
