# Supplementary material for: Assessing the endocrine disrupting potentials and genotoxicity in environmental samples from Taiwanese rivers
Source: Genes Environ. 2019 Dec 30;41:24. doi: 10.1186/s41021-019-0140-9 (PMC6937667; doi:10.1186/s41021-019-0140-9)
Supplement: Supplementary file 1 — Additional file 1: Table S1. Basic information of Yanshuei River, Erren River, and Agondian River*1. Table S2. Biological parameters of the fish captured at downstream of Yanshuei River, Erren River, and Agondian River. Table S3. Method detection limits (MDLs) and recoveries of target PAHs investigated in this study. Figure S1. Concentration-activity curves of agonist/antagonist compounds for AR (DHT/FLU), ER (17β-E2/OHT), RXR (9cRA), and TR (T3). Figure S2. (A) AR (B) ER (C) RXR (D) TR agonist/antagonist activities elicited by 10−/5-fold concentrated dry-season river water extracts (Water-D) and wet-season river water extracts (Water-W). Figure S3. Concentration-survival rates of Rec+ and Rec– strains exposed to genotoxic standard 4-NQO and dry−/wet-season (D/W) sediment extracts of E1, E7, A1, and A2. [file 41021_2019_140_MOESM1_ESM.pdf]

SUPPLEMENTARY MATERIAL

Assessing the endocrine disrupting potentials and genotoxicity in environmental samples from Taiwanese rivers

Pei-Hsin Chou[1*], Chien-Hsun Chen[1], Kuang-Yu Chen[1], Fung-Chi Ko[2], Tsung-Ya Tsai[1], Yi-Po Yeh[1]

[1] *Department of Environmental Engineering, National Cheng Kung University, 1, University Road, Tainan, 70101, Taiwan*

[2] *Graduate institute of Marine Biology, National Dong Hwa University, National Museum of Marine Biology and Aquarium, 2, Houwan Road, Pingtung, 944, Taiwan*

[*] Corresponding author. *E-mail address*: phchou@mail.ncku.edu.tw; Phone: +886 6 2757575 ext.65840; Fax: +886 6 2752790.

Table S1 Basic information of Yanshuei River, Erren River, and Agondian River[*1]

River	Length (km)	Catchment (km^2)	Population (×1000)	BOD Load ($kg\ d^{-1}$)
Yanshuei	41.3	343.2	556	32599 (~63% from domestic wastewater)
Erren	61.2	339.2	365	22607 (~66% from domestic wastewater)
Agondian	38	137	135	12188 (~48% from swine wastewater)

*1 EPA (Environmental Protection Administration, Taiwan), 2013. Strategy and Implementation of River Rehabilitation and Water Quality Improvement in Southern Taiwan (in Chinese).

Table S2 Biological parameters of the fish captured at downstream of Yanshuei River, Erren River, and Agondian River

Fish	Species	Weight (g)	Length (cm)	Muscle weight (g)		Lipid content
				Wet wt.	Dry wt.	
Y5F1	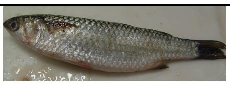 *Mugil cephalus*	163.8	22	2.86	0.72	14.1%
Y5F2	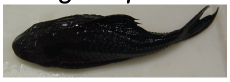 *Pterygoplichthys pardalis*	111.6	18	2.45	0.46	4.4%
Y5F3	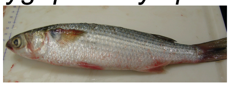 *Mugil cephalus*	208.2	25	2.00	0.48	17.4%
Y5F4	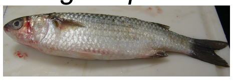 *Mugil cephalus*	229.4	24	3.21	0.79	12.3%
E4F1	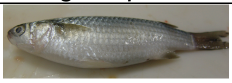 *Mugil cephalus*	56.2	15	0.74	0.16	6.7%
E4F2	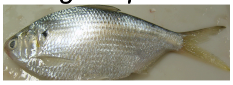 *Nematalosa come*	89.1	21	1.94	0.39	6.0%
A3F1	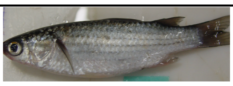 *Mugil cephalus*	46.4	15	1.40	0.31	2.7%
A3F2	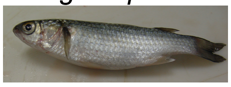 *Mugil cephalus*	62.6	17	2.12	0.36	7.8%
A3F3	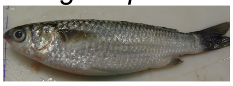 *Mugil cephalus*	114.6	21	1.18	0.25	8.0%
A3F4	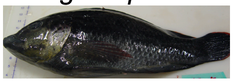 *Oreochromis mossambicus*	252.7	24	1.91	0.11	9.6%

Table S3 Method detection limits (MDLs) and recoveries of target PAHs investigated in this study

Substance	MDL (ng)	Recovery	Substance	MDL (ng)	Recovery
Naphthalene	13.31	133%	Fluoranthene	2.28	94%
2-Methylnaphthalene	1.56	88%	2,3-Dimethylanthracene	0.12	95%
1-Methylnaphthalene	1.22	87%	Pyrene	1.97	95%
2,6-Dimethylnaphthalene	0.89	95%	9,10-Dimethylanthracene	4.67	83%
1,3-Dimethylnaphthalene	0.73	94%	2-Methylfluoranthene	0.20	103%
1,6-Dimethylnaphthalene	0.38	97%	Retene	0.38	101%
1,4-Dimethylnaphthalene	0.41	110%	Benzo[*a*]fluorene	0.36	96%
1,5-Dimethylnaphthalene	0.14	88%	Benzo[*b*]fluorene	0.13	98%
Acenapthylene	0.15	94%	1-Methylpyrene	0.23	103%
1,2-Dimethylnaphthalene	0.21	97%	Benz[*a*]anthracene	0.22	107%
1,8-Dimethylnaphthalene	7.3	100%	Chrysene + Triphenylene	0.36	53%
Acenaphthene	0.51	99%	1-Methylbenz[*a*]anthracene	0.05	108%
2,3,5-Trimethylnaphthale	0.08	70%	4-/6-Methylchrysene	0.84	107%
Fluorene	1.6	79%	3,9-Dimethylbenz[*a*]anthracene	0.04	110%
1-Methylfuorene	0.47	80%	Benzo[*b*]fluoranthene	0.35	113%
Dibenzothiophene	1.24	86%	Benzo[*k*]fluoranthene	0.16	113%
Phenanthrene	7.99	105%	Benzo[e]pyrene	0.55	112%
Anthracene	0.46	90%	Benzo[*a*]pyrene	0.14	110%
2-Methylphenanthrene	1.43	103%	Perylene	0.24	107%
2-Methylanthracene	0.25	95%	10-Methylbenzo[*a*]pyrene	0.10	115%
4,5-Methylenephenanthrene	0.44	93%	7,10-Dimethylbenzo[*a*]pyrene	0.16	90%
1-Methylanthracene	0.62	95%	Indeno[1,2,3-*c*,*d*]pyrene	0.12	104%
1-Methylphenanthrene	0.74	168%	Dibenz[*a*,*h*]anthracene	0.15	91%
4,6-Dimethyldibenzothiophene	0.44	131%	Benzo[*g*,*h*,*i*]perylene	0.14	93%
9-Methylanthracene	0.14	67%	Coronene	0.09	109%
3,6-Dimethylphenanthrene	0.04	95%			

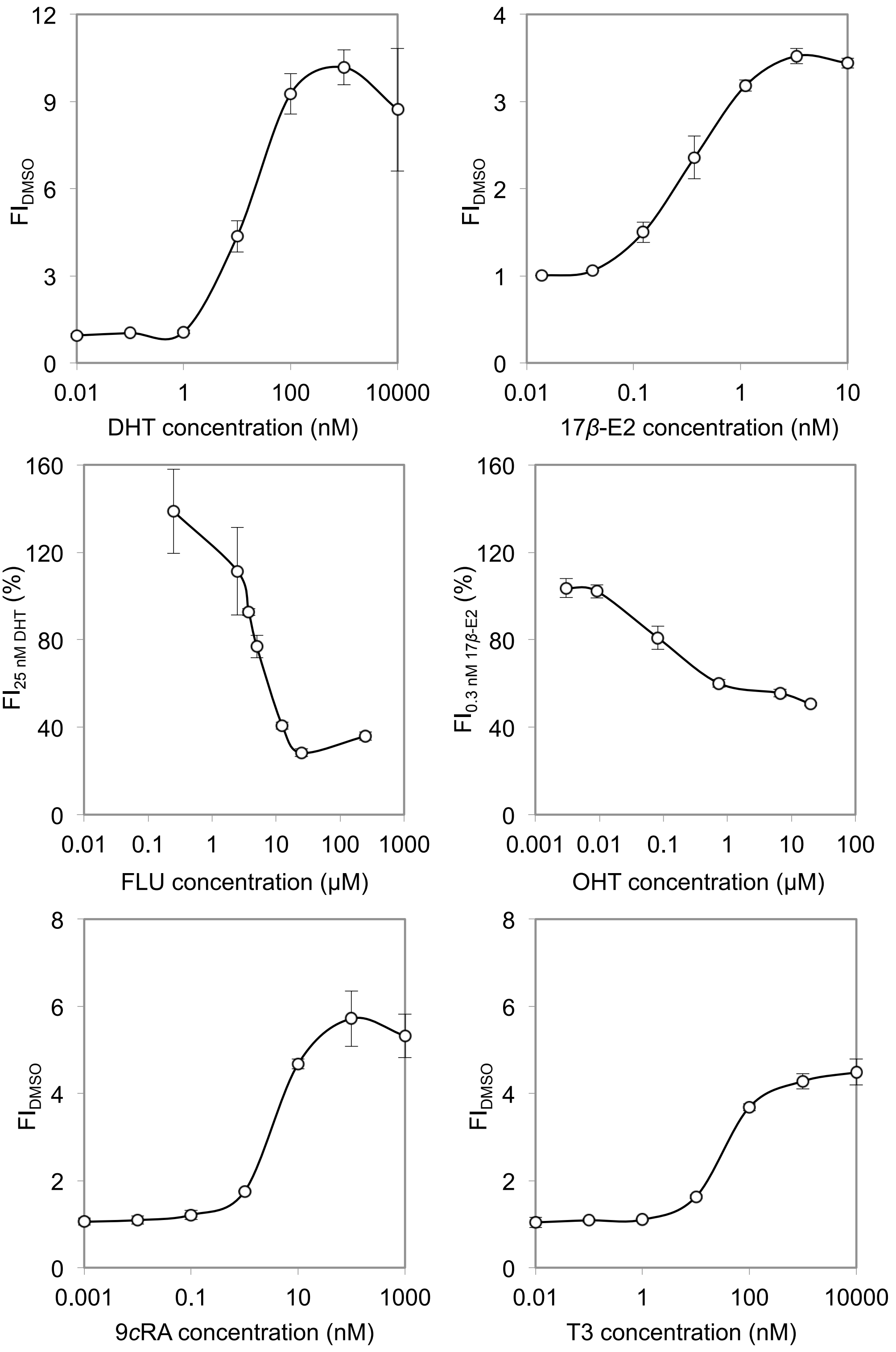

Figure S1 Concentration-activity curves of agonist/antagonist compounds for AR (DHT/FLU), ER (17*β*-E2/OHT), RXR (9*c*RA), and TR (T3).

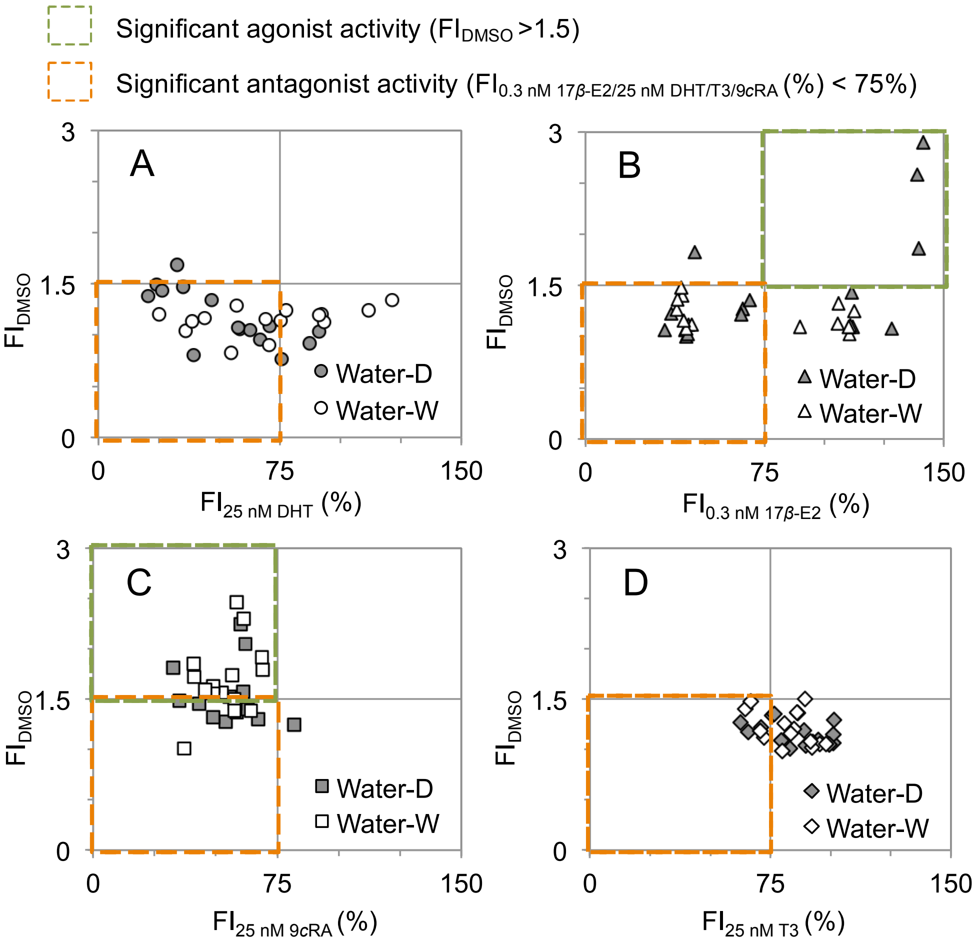

Figure S2 (A) AR (B) ER (C) RXR (D) TR agonist/antagonist activities elicited by 10-/5-fold concentrated dry-season river water extracts (Water-D) and wet-season river water extracts (Water-W).

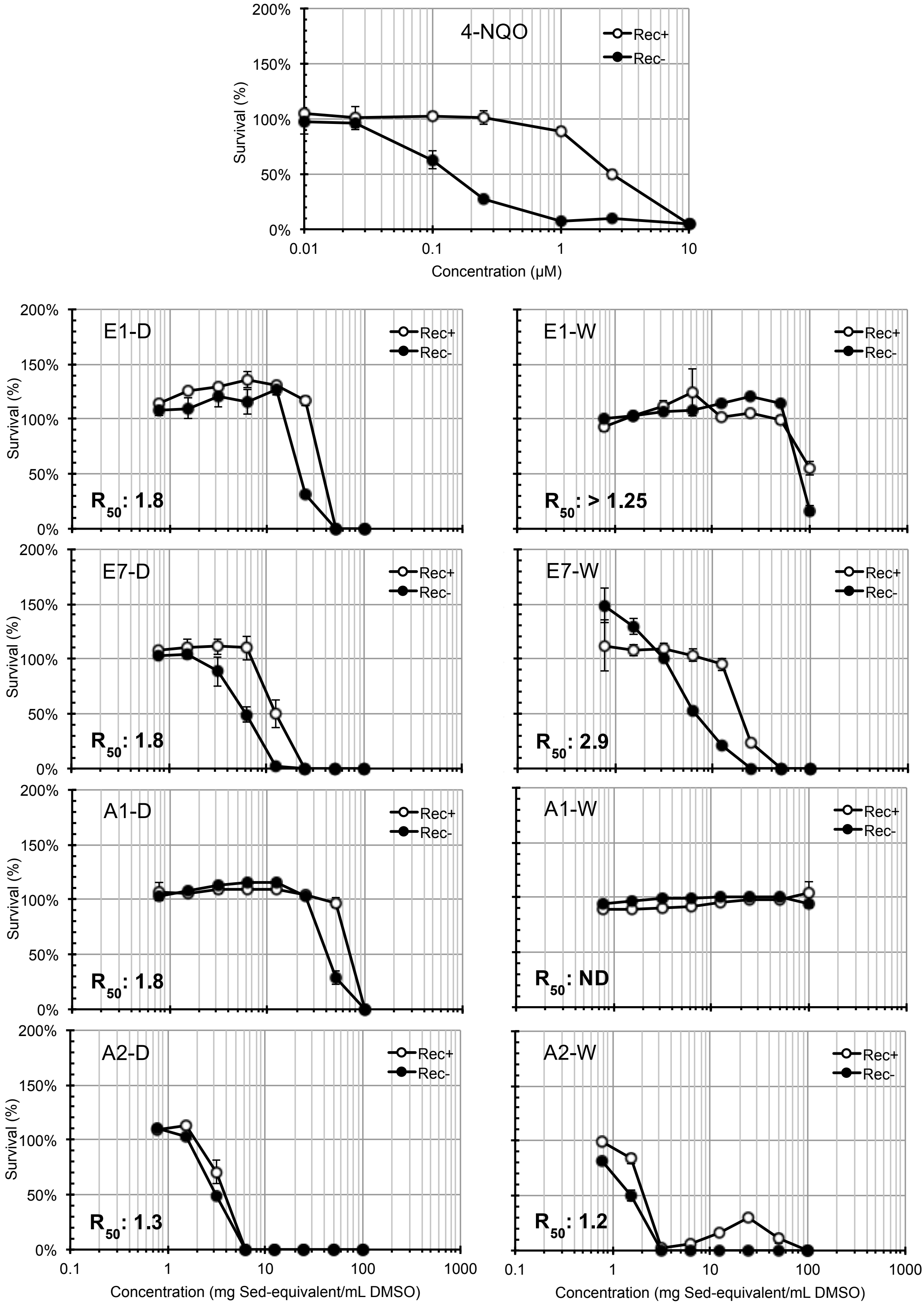

Figure S3 Concentration-survival rates of Rec+ and Rec– strains exposed to genotoxic standard 4-NQO and dry-/wet-season (D/W) sediment extracts of E1, E7, A1, and A2.
